# *trans*-Dichloridobis{2-chloro-6-[(3-fluoro­benz­yl)amino]-9-isopropyl-9*H*-purine-κ*N* ^7^}platinum(II)

**DOI:** 10.1107/S1600536813013202

**Published:** 2013-05-18

**Authors:** Zdeněk Trávníček, Pavel Štarha

**Affiliations:** a Department of Inorganic Chemistry, Faculty of Science, Palacký University, 17. listopadu 12, CZ-771 46 Olomouc, Czech Republic

## Abstract

In the title compound, *trans*-[PtCl_2_(C_15_H_15_ClFN_5_)_2_], the Pt^II^ atom, located on an inversion centre, is coordinated by the purine N atoms of the 2-chloro-6-[(3-fluoro­benz­yl)amino]-9-isopropyl-9*H*-purine ligands and two Cl atoms in a slightly distorted *trans*-square-planar coordination geometry [N—Pt—Cl angles = 89.91 (5) and 90.09 (5)°]. Weak intra­molecular N—H⋯Cl contacts occur. In the crystal, C—H⋯Cl and C—H⋯F contacts, as well as weak π–π stacking inter­actions [centroid–centroid distances = 3.5000 (11) and 3.6495 (12) Å] connect the mol­ecules into a three-dimensional architecture.

## Related literature

For the structures of platinum(II) dichlorido complexes involving different 2-chloro-6-[(substituted-benz­yl)amino]-9-isopropyl-9*H*-purine derivatives, see: Trávníček *et al.* (2006 ▶); Szüčová *et al.* (2008 ▶). For the synthesis of 2-chloro-6-[(substituted-benzyl)amino]-9-isopropyl-9H-purine derivatives, see: Štarha *et al.* (2009 ▶).
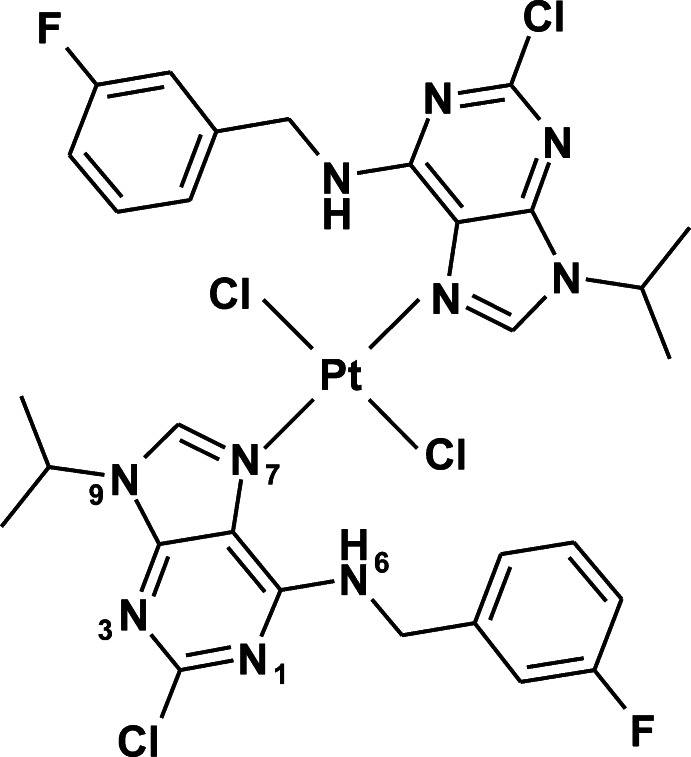


## Experimental

### 

#### Crystal data

[PtCl_2_(C_15_H_15_ClFN_5_)_2_]
*M* *_r_* = 905.53
Monoclinic, 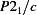

*a* = 9.37786 (13) Å
*b* = 12.86530 (17) Å
*c* = 14.2891 (2) Å
β = 107.9165 (16)°
*V* = 1640.36 (4) Å^3^
*Z* = 2
Mo *K*α radiation
μ = 4.65 mm^−1^
*T* = 105 K
0.35 × 0.35 × 0.35 mm

#### Data collection

Agilent Xcalibur Sapphire2 diffractometer
Absorption correction: multi-scan (*CrysAlis PRO*; Agilent, 2012 ▶) *T*_min_ = 0.293, *T*_max_ = 0.293
13582 measured reflections
2881 independent reflections
2726 reflections with *I* > 2σ(*I*)
*R*_int_ = 0.010

#### Refinement

*R*[*F* ^2^ > 2σ(*F* ^2^)] = 0.015
*wR*(*F* ^2^) = 0.037
*S* = 1.10
2881 reflections
216 parameters
H-atom parameters constrained
Δρ_max_ = 0.52 e Å^−3^
Δρ_min_ = −0.32 e Å^−3^

### 

Data collection: *CrysAlis PRO* (Agilent, 2012 ▶); cell refinement: *CrysAlis PRO*; data reduction: *CrysAlis PRO*; program(s) used to solve structure: *SHELXS97* (Sheldrick, 2008 ▶); program(s) used to refine structure: *SHELXL97* (Sheldrick, 2008 ▶); molecular graphics: *DIAMOND* (Brandenburg, 2011 ▶); software used to prepare material for publication: *publCIF* (Westrip, 2010 ▶).

## Supplementary Material

Crystal structure: contains datablock(s) I, global. DOI: 10.1107/S1600536813013202/jj2166sup1.cif

Structure factors: contains datablock(s) I. DOI: 10.1107/S1600536813013202/jj2166Isup2.hkl

Additional supplementary materials:  crystallographic information; 3D view; checkCIF report

## Figures and Tables

**Table 1:** Hydrogen-bond geometry (Å, °)

*D*—H⋯*A*	*D*—H	H⋯*A*	*D*⋯*A*	*D*—H⋯*A*
N6—H6*A*⋯Cl2^i^	0.88	2.53	3.222 (2)	136
C13—H13*A*⋯Cl2^ii^	0.95	2.86	3.492 (2)	125
C18—H18*A*⋯F1^iii^	0.98	2.49	3.450 (3)	166

Symmetry codes: (i) 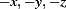; (ii) 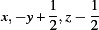; (iii) 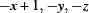.

**Fig. 1.**
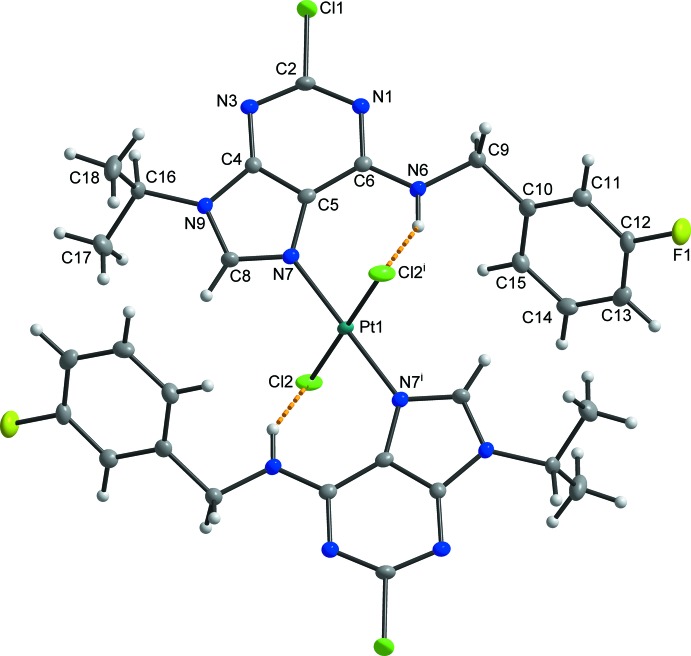
The molecular structure of the title compound with the atom numbering scheme and the non-hydrogen atoms at the 50% probability level. Dashed lines indicate weak N6—H6A···Cl2i intramolecular interactions (symmetry code: i) -x, -y, -z).

**Fig. 2.**
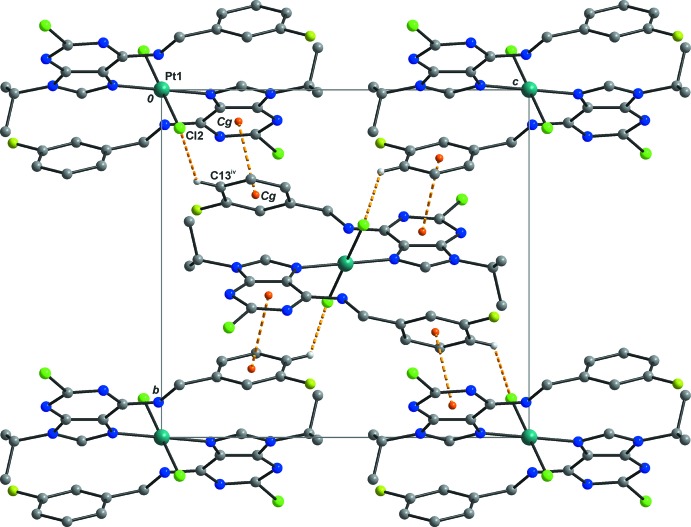
Packing diagram of the title compound viewed along the a axis. Dashed lines indicate weak C13—H13Aiv···Cl2 intermolecular and π···π stacking interactions (Cg···Cg = 3.5001 Å) (symmetry code: iv) x, -y+1/2, z + 1/2).

**Fig. 3.**
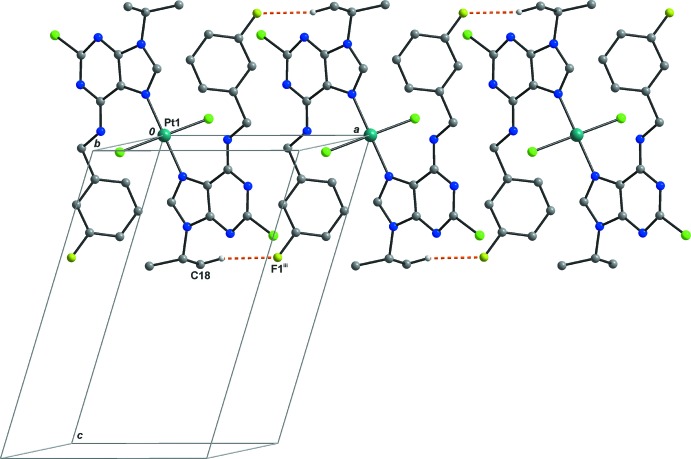
Packing diagram of the title compound viewed along the b axis. Dashed lines indicate weak C18—H18A···F1iii intermolecular interactions (symmetry code: iii) -x+1, -y, -z).

## supplementary crystallographic information

### Comment

In the title compound, the Pt^II^ atom is located on an inversion centre and thus, the asymmetric unit contains one-half of the described platinum(II) complex (Fig. 1). The central Pt^II^ atom is four-coordinated by two chloride anions [Pt—Cl = 2.2940 (5) Å] and two 2-chloro-6-[(3-fluorobenzyl)amino]-9-isopropyl-9*H*-purine molecules [Pt—N = 2.011 (2) Å], which are bonded to platinum through their N7 atoms of the purine moieties. The geometry of the complex is slightly distorted square-planar with the N—Pt—Cl angles in the vicinity of the central metal atom of 89.91 (5)° and 90.09 (5)°, and the mean plane fitted through the PtCl_2_N_2_ unit (r.m.s. deviation = 0.000 Å) being planar. Both the essentially planar purine moieties [with the maximum deviation of 0.050 (2) Å for the C5 atom] are mutually coplanar and each of them forms the dihedral angle of 62.04 (3)° and 49.58 (5)° with the PtCl_2_N_2_ unit, and the benzene ring, respectively (Fig. 1). The molecular structure involves weak N6—H6A···Cl2 intramolecular interactions (Table 1, Fig. 1). In the crystal, the molecules are connected together through weak C13—H13A···Cl2, C18—H18A···F1 and π···π (between the six-membered pyrimidine and benzene rings) intermolecular interactions into a three-dimensional architecture (Fig. 2 and 3, Table 1).

### Experimental

The solution of 2-chloro-6-[(3-fluorobenzyl)amino]-9-isopropyl-9*H*-purine (0.5 mmol; prepared according to the previously described procedure, (Štarha *et al.*, 2009) in acetone (10 ml) was slowly poured into the distilled water solution of K_2_PtCl_4_ (0.25 mmol). The reaction mixture was stirred at laboratory temperature, until the initial orange colour turned to yellow. The solid was collected by filtration and washed with distilled water and acetone. Part of the product was recrystallized from *N,N`*-dimethylformamide. The crystals suitable for a single-crystal X-ray analysis formed after two weeks. Analysis calculated for C_30_H_30_N_10_Cl_4_F_2_Pt_1_: C 39.8, H 3.3, N 15.5%; found: C 39.9, H 3.3, N 15.3%. Elemental analysis (C, H, N) was performed on a Thermo Scientific Flash 2000 CHNO-S Analyzer.

### Refinement

Non-hydrogen atoms were refined anisotropically and hydrogen atoms were located in difference maps and refined using the riding model with C—H = 0.95 (CH), C—H = 0.99 (CH_2_), C—H = 0.98 (CH_3_) Å, and N—H = 0.88 Å, with *U*_iso_(H) = 1.2*U*_eq_(CH, CH_2_, NH) and 1.5*U*_eq_(CH_3_). The maximum and minimum residual electron density peaks of 0.52 and -0.32 e Å^-3^ were located 0.87 Å, and 0.27 Å from the Pt1, and H6A atoms, respectively.

### Crystal data

**Table:**

[PtCl_2_(C_15_H_15_ClFN_5_)_2_]	*F*(000) = 888
*M**_r_* = 905.53	*D*_x_ = 1.833 Mg m^−^^3^
Monoclinic, *P*2_1_/*c*	Mo *K*α radiation, λ = 0.71073 Å
Hall symbol: -P 2ybc	Cell parameters from 15658 reflections
*a* = 9.37786 (13) Å	θ = 3.0–31.7°
*b* = 12.86530 (17) Å	µ = 4.65 mm^−^^1^
*c* = 14.2891 (2) Å	*T* = 105 K
β = 107.9165 (16)°	Prism, yellow-orange
*V* = 1640.36 (4) Å^3^	0.35 × 0.35 × 0.35 mm
*Z* = 2	

### Data collection

**Table:**

Agilent Xcalibur Sapphire2 diffractometer	2881 independent reflections
Radiation source: Enhance (Mo) X-ray Source	2726 reflections with *I* > 2σ(*I*)
Graphite monochromator	*R*_int_ = 0.010
Detector resolution: 8.3611 pixels mm^-1^	θ_max_ = 25.0°, θ_min_ = 3.0°
ω scans	*h* = −11→11
Absorption correction: multi-scan (*CrysAlis PRO*; Agilent, 2012)	*k* = −15→13
*T*_min_ = 0.293, *T*_max_ = 0.293	*l* = −16→16
13582 measured reflections	

### Refinement

**Table:**

Refinement on *F*^2^	Primary atom site location: structure-invariant direct methods
Least-squares matrix: full	Secondary atom site location: difference Fourier map
*R*[*F*^2^ > 2σ(*F*^2^)] = 0.015	Hydrogen site location: inferred from neighbouring sites
*wR*(*F*^2^) = 0.037	H-atom parameters constrained
*S* = 1.10	*w* = 1/[σ^2^(*F*_o_^2^) + (0.0204*P*)^2^ + 1.5464*P*] where *P* = (*F*_o_^2^ + 2*F*_c_^2^)/3
2881 reflections	(Δ/σ)_max_ < 0.001
216 parameters	Δρ_max_ = 0.52 e Å^−^^3^
0 restraints	Δρ_min_ = −0.32 e Å^−^^3^

### Special details

**Table:**

Geometry. All e.s.d.'s (except the e.s.d. in the dihedral angle between two l.s. planes) are estimated using the full covariance matrix. The cell e.s.d.'s are taken into account individually in the estimation of e.s.d.'s in distances, angles and torsion angles; correlations between e.s.d.'s in cell parameters are only used when they are defined by crystal symmetry. An approximate (isotropic) treatment of cell e.s.d.'s is used for estimating e.s.d.'s involving l.s. planes.
Refinement. Refinement of *F*^2^ against ALL reflections. The weighted *R*-factor *wR* and goodness of fit *S* are based on *F*^2^, conventional *R*-factors *R* are based on *F*, with *F* set to zero for negative *F*^2^. The threshold expression of *F*^2^ > σ(*F*^2^) is used only for calculating *R*-factors(gt) *etc*. and is not relevant to the choice of reflections for refinement. *R*-factors based on *F*^2^ are statistically about twice as large as those based on *F*, and *R*- factors based on ALL data will be even larger.

### Fractional atomic coordinates and isotropic or equivalent isotropic displacement parameters (Å^2^)

**Table:**

	*x*	*y*	*z*	*U*_iso_*/*U*_eq_	
Pt1	0.0000	0.0000	0.0000	0.01222 (5)	
Cl1	0.73503 (6)	0.18413 (6)	0.31702 (4)	0.03616 (16)	
Cl2	−0.15533 (5)	0.11059 (4)	0.04896 (4)	0.02297 (12)	
F1	0.32167 (15)	0.15374 (11)	−0.40416 (9)	0.0297 (3)	
N1	0.52347 (19)	0.13487 (14)	0.15853 (12)	0.0174 (4)	
N3	0.49172 (18)	0.08828 (14)	0.31434 (12)	0.0164 (4)	
N6	0.34612 (19)	0.10095 (14)	0.00859 (12)	0.0176 (4)	
H6A	0.2599	0.0723	−0.0239	0.021*	
N7	0.1537 (2)	0.01264 (12)	0.13330 (14)	0.0145 (4)	
N9	0.2482 (2)	0.01119 (12)	0.29616 (14)	0.0148 (4)	
C2	0.5616 (2)	0.12816 (17)	0.25559 (15)	0.0189 (4)	
C4	0.3566 (2)	0.05155 (15)	0.26050 (14)	0.0137 (4)	
C5	0.2990 (2)	0.05145 (15)	0.15906 (14)	0.0137 (4)	
C6	0.3894 (2)	0.09529 (15)	0.10649 (14)	0.0140 (4)	
C8	0.1290 (3)	−0.01030 (15)	0.21725 (17)	0.0160 (4)	
H8A	0.0377	−0.0387	0.2215	0.019*	
C9	0.4310 (2)	0.15101 (17)	−0.04837 (15)	0.0187 (4)	
H9A	0.5135	0.1049	−0.0524	0.022*	
H9B	0.4755	0.2165	−0.0159	0.022*	
C10	0.3281 (2)	0.17380 (15)	−0.15035 (15)	0.0157 (4)	
C11	0.3731 (2)	0.15135 (16)	−0.23221 (15)	0.0179 (4)	
H11A	0.4677	0.1201	−0.2251	0.021*	
C12	0.2768 (2)	0.17560 (16)	−0.32396 (15)	0.0179 (4)	
C13	0.1394 (2)	0.22057 (16)	−0.33922 (15)	0.0205 (4)	
H13A	0.0766	0.2364	−0.4037	0.025*	
C14	0.0949 (2)	0.24231 (17)	−0.25725 (16)	0.0209 (4)	
H14A	0.0000	0.2733	−0.2652	0.025*	
C15	0.1886 (2)	0.21895 (16)	−0.16387 (15)	0.0191 (4)	
H15A	0.1569	0.2340	−0.1083	0.023*	
C16	0.2665 (3)	−0.01102 (16)	0.40122 (16)	0.0181 (5)	
H16A	0.3434	0.0378	0.4424	0.022*	
C17	0.1208 (3)	0.00727 (17)	0.42384 (19)	0.0238 (5)	
H17A	0.0852	0.0780	0.4042	0.036*	
H17B	0.1375	−0.0014	0.4945	0.036*	
H17C	0.0455	−0.0429	0.3874	0.036*	
C18	0.3242 (3)	−0.1208 (2)	0.42492 (17)	0.0307 (5)	
H18A	0.4155	−0.1299	0.4062	0.046*	
H18B	0.2477	−0.1702	0.3883	0.046*	
H18C	0.3466	−0.1333	0.4956	0.046*	

### Atomic displacement parameters (Å^2^)

**Table:**

	*U*^11^	*U*^22^	*U*^33^	*U*^12^	*U*^13^	*U*^23^
Pt1	0.00955 (7)	0.01405 (7)	0.01116 (7)	−0.00130 (4)	0.00039 (5)	−0.00091 (4)
Cl1	0.0192 (3)	0.0674 (5)	0.0179 (3)	−0.0222 (3)	−0.0002 (2)	0.0010 (3)
Cl2	0.0140 (2)	0.0248 (3)	0.0274 (3)	0.0009 (2)	0.0023 (2)	−0.0114 (2)
F1	0.0371 (8)	0.0390 (8)	0.0154 (6)	0.0029 (6)	0.0115 (6)	−0.0034 (6)
N1	0.0135 (8)	0.0234 (9)	0.0147 (8)	−0.0018 (7)	0.0034 (7)	0.0004 (7)
N3	0.0122 (8)	0.0219 (9)	0.0140 (8)	−0.0012 (7)	0.0022 (7)	−0.0011 (7)
N6	0.0149 (8)	0.0232 (9)	0.0123 (8)	−0.0060 (7)	0.0008 (7)	0.0012 (7)
N7	0.0133 (9)	0.0159 (9)	0.0130 (9)	−0.0009 (6)	0.0022 (7)	−0.0001 (6)
N9	0.0141 (9)	0.0180 (9)	0.0112 (9)	−0.0013 (6)	0.0024 (7)	0.0007 (6)
C2	0.0112 (9)	0.0260 (12)	0.0174 (10)	−0.0028 (8)	0.0010 (8)	−0.0011 (9)
C4	0.0133 (9)	0.0137 (10)	0.0140 (10)	0.0016 (8)	0.0039 (8)	0.0006 (8)
C5	0.0124 (9)	0.0125 (10)	0.0145 (9)	0.0013 (8)	0.0015 (8)	−0.0010 (8)
C6	0.0128 (9)	0.0133 (10)	0.0148 (10)	0.0016 (8)	0.0026 (8)	−0.0006 (8)
C8	0.0125 (11)	0.0189 (11)	0.0155 (11)	−0.0032 (8)	0.0024 (9)	−0.0003 (8)
C9	0.0155 (10)	0.0247 (11)	0.0158 (10)	−0.0026 (9)	0.0046 (8)	0.0018 (9)
C10	0.0166 (10)	0.0139 (10)	0.0163 (10)	−0.0030 (8)	0.0046 (8)	0.0005 (8)
C11	0.0177 (10)	0.0170 (10)	0.0192 (10)	0.0007 (8)	0.0059 (8)	0.0010 (8)
C12	0.0247 (11)	0.0170 (10)	0.0138 (10)	−0.0026 (9)	0.0085 (9)	−0.0006 (8)
C13	0.0222 (11)	0.0191 (11)	0.0169 (10)	−0.0020 (9)	0.0009 (8)	0.0044 (8)
C14	0.0148 (10)	0.0200 (11)	0.0268 (11)	0.0022 (8)	0.0050 (9)	0.0051 (9)
C15	0.0214 (11)	0.0196 (11)	0.0187 (10)	0.0007 (9)	0.0098 (9)	0.0009 (9)
C16	0.0164 (11)	0.0283 (12)	0.0097 (10)	−0.0043 (8)	0.0039 (9)	−0.0014 (8)
C17	0.0232 (13)	0.0290 (13)	0.0227 (13)	−0.0019 (9)	0.0121 (10)	−0.0015 (9)
C18	0.0334 (13)	0.0408 (14)	0.0195 (11)	0.0122 (11)	0.0103 (10)	0.0117 (10)

### Geometric parameters (Å, º)

**Table:**

Pt1—N7	2.0108 (18)	C9—H9A	0.9900
Pt1—N7^i^	2.0109 (18)	C9—H9B	0.9900
Pt1—Cl2	2.2940 (5)	C10—C15	1.390 (3)
Pt1—Cl2^i^	2.2940 (5)	C10—C11	1.390 (3)
Cl1—C2	1.748 (2)	C11—C12	1.380 (3)
F1—C12	1.366 (2)	C11—H11A	0.9500
N1—C2	1.324 (3)	C12—C13	1.368 (3)
N1—C6	1.348 (3)	C13—C14	1.387 (3)
N3—C2	1.317 (3)	C13—H13A	0.9500
N3—C4	1.349 (3)	C14—C15	1.386 (3)
N6—C6	1.333 (3)	C14—H14A	0.9500
N6—C9	1.453 (3)	C15—H15A	0.9500
N6—H6A	0.8800	C16—C18	1.513 (3)
N7—C8	1.323 (3)	C16—C17	1.516 (3)
N7—C5	1.390 (3)	C16—H16A	1.0000
N9—C8	1.350 (3)	C17—H17A	0.9800
N9—C4	1.372 (3)	C17—H17B	0.9800
N9—C16	1.485 (3)	C17—H17C	0.9800
C4—C5	1.382 (3)	C18—H18A	0.9800
C5—C6	1.412 (3)	C18—H18B	0.9800
C8—H8A	0.9500	C18—H18C	0.9800
C9—C10	1.508 (3)		
			
N7—Pt1—N7^i^	180.0	H9A—C9—H9B	108.3
N7—Pt1—Cl2	89.91 (5)	C15—C10—C11	119.10 (19)
N7^i^—Pt1—Cl2	90.09 (5)	C15—C10—C9	120.70 (18)
N7—Pt1—Cl2^i^	90.09 (5)	C11—C10—C9	120.19 (18)
N7^i^—Pt1—Cl2^i^	89.91 (5)	C12—C11—C10	118.25 (19)
Cl2—Pt1—Cl2^i^	180.00 (3)	C12—C11—H11A	120.9
C2—N1—C6	117.27 (17)	C10—C11—H11A	120.9
C2—N3—C4	109.68 (17)	C13—C12—F1	118.13 (19)
C6—N6—C9	124.74 (17)	C13—C12—C11	123.80 (19)
C6—N6—H6A	117.6	F1—C12—C11	118.06 (19)
C9—N6—H6A	117.6	C12—C13—C14	117.65 (19)
C8—N7—C5	105.71 (18)	C12—C13—H13A	121.2
C8—N7—Pt1	124.49 (15)	C14—C13—H13A	121.2
C5—N7—Pt1	129.68 (14)	C15—C14—C13	120.2 (2)
C8—N9—C4	106.56 (18)	C15—C14—H14A	119.9
C8—N9—C16	127.83 (19)	C13—C14—H14A	119.9
C4—N9—C16	125.45 (18)	C14—C15—C10	121.01 (19)
N3—C2—N1	131.76 (19)	C14—C15—H15A	119.5
N3—C2—Cl1	114.12 (15)	C10—C15—H15A	119.5
N1—C2—Cl1	114.11 (15)	N9—C16—C18	109.15 (17)
N3—C4—N9	126.44 (18)	N9—C16—C17	110.76 (19)
N3—C4—C5	126.43 (18)	C18—C16—C17	112.39 (19)
N9—C4—C5	107.08 (17)	N9—C16—H16A	108.1
C4—C5—N7	108.25 (17)	C18—C16—H16A	108.1
C4—C5—C6	116.90 (17)	C17—C16—H16A	108.1
N7—C5—C6	134.64 (18)	C16—C17—H17A	109.5
N6—C6—N1	119.28 (18)	C16—C17—H17B	109.5
N6—C6—C5	122.78 (18)	H17A—C17—H17B	109.5
N1—C6—C5	117.92 (17)	C16—C17—H17C	109.5
N7—C8—N9	112.38 (19)	H17A—C17—H17C	109.5
N7—C8—H8A	123.8	H17B—C17—H17C	109.5
N9—C8—H8A	123.8	C16—C18—H18A	109.5
N6—C9—C10	109.24 (16)	C16—C18—H18B	109.5
N6—C9—H9A	109.8	H18A—C18—H18B	109.5
C10—C9—H9A	109.8	C16—C18—H18C	109.5
N6—C9—H9B	109.8	H18A—C18—H18C	109.5
C10—C9—H9B	109.8	H18B—C18—H18C	109.5

Symmetry code: (i) −*x*, −*y*, −*z*.

### Hydrogen-bond geometry (Å, º)

**Table:**

*D*—H···*A*	*D*—H	H···*A*	*D*···*A*	*D*—H···*A*
N6—H6*A*···Cl2^i^	0.88	2.53	3.222 (2)	136
C13—H13*A*···Cl2^ii^	0.95	2.86	3.492 (2)	125
C18—H18*A*···F1^iii^	0.98	2.49	3.450 (3)	166

Symmetry codes: (i) −*x*, −*y*, −*z*; (ii) *x*, −*y*+1/2, *z*−1/2; (iii) −*x*+1, −*y*, −*z*.
